# Association between increased microbiota awareness and lower gastrointestinal symptom severity: a cross-sectional analysis

**DOI:** 10.1186/s12876-026-05077-w

**Published:** 2026-07-07

**Authors:** Kadriye Toprak, Dilara Nur Kaplan, Ezgi Nur Cinar, Gozde Senturk, Hilal Eravci, Merve Oksuz Dag, Zeyneb Yildirim, Feride Ayyildiz

**Affiliations:** 1 Department of Nutrition and Dietetics, Faculty of Health Sciences, Ankara Medipol University, Altındağ, Ankara, 06050 Turkey; 2 Department of Nutrition and Dietetics, Faculty of Health Sciences, Karabuk University, Karabuk, Turkey; 3 Department of Nutrition and Dietetics, Department of Athlete Health and Performance, Ministry of Youth and Sports, Ankara, Turkey; 4 Department of Nutrition and Dietetics, Institute of Health Sciences, Ankara Medipol University, Ankara, Turkey; 5 Department of Nutrition and Dietetics, School of Health Sciences, Gazi University, Ankara, Turkey

**Keywords:** Microbiota awareness level, Probiotic food consumption frequency, Gastrointestinal symptom severity, Gut microbiota

## Abstract

**Background:**

Today, gastrointestinal symptoms represent a significant health concern, affecting not only the digestive system but also overall health and quality of life. The growing awareness of the role of microbiota in health has increased the interest in research into the connection between probiotic food consumption and gastrointestinal health. This study aimed to examine the association between microbiota awareness level and probiotic-containing food consumption frequency and gastrointestinal symptoms.

**Methods:**

This cross-sectional study was conducted with 642 participants. The consumption habits and frequency of intake of various foods containing probiotics were investigated. Additionally, the Microbiota Awareness Scale assessed individuals' microbiota awareness levels, while gastrointestinal symptoms were assessed with the Gastrointestinal Symptom Rating Scale (GSRS). The associations among microbiota awareness score, probiotic-containing food consumption frequency, and some basic independent variables and GSRS were evaluated using hierarchical linear regression analysis.

**Results:**

Microbiota awareness score was found to be significantly associated with the GSRS total score (β = -0.288;95% CI:-0.394 to -0.243; *p* < 0.001). Age (*p* = 0.001), body mass index (*p* = 0.044), gender (*p* < 0.001), educational status (*p* = 0.035), and presence of digestive system problems (*p* < 0.001) were found to have significant independent associations with GSRS scores.

**Conclusions:**

Microbiota awareness level remained an independent correlate of GSRS after controlling for age, body mass index, and gender. These findings suggest that higher microbiota awareness is associated with lower gastrointestinal symptom severity.

## Background

The gut microbiota is a complex ecosystem that harbours at least 1,000 different species and trillions of microorganisms [1]. This ecosystem consists primarily of bacteria belonging to the Firmicutes and Bacteroidetes phyla, but also includes other microbial commensals such as archaea, viruses, fungi, and protozoa [2]. The composition of the gut microbiota plays a decisive role in the host's metabolic homeostasis, immune system development, neurological functions, and overall health [3, 4]. The gut microbiota generally has a stable ecosystem; it can re-establish homeostasis following disruptions caused by unhealthy diets, antibiotics, medications, pathogens, and other factors. However, under the prolonged influence of chronic stress, poor nutrition, antibiotic use, pathogenic infections, and other environmental factors, a pathophysiological condition known as ‘dysbiosis’ arises when the microbial composition is permanently disrupted [3]. Dysbiosis is characterised by a decrease in microbial diversity, a reduction in the number of beneficial commensals, and an overgrowth of potentially pathogenic species. Numerous clinical and experimental studies have demonstrated that gut microbiota dysbiosis is associated with irritable bowel syndrome, inflammatory bowel disease, obesity, type 2 diabetes, metabolic syndrome, cardiovascular diseases, neurodegenerative diseases, and even certain types of cancer [4–6]. Therefore, preserving a healthy gut microbiota is important for overall health.

Probiotics are defined as live microorganisms that, when administered in adequate amounts, confer a health benefit on the host by improving the characteristics of the endogenous microflora [7]. These health benefits extend beyond basic gut health to include broader systemic effects such as immunomodulation, reduction of oxidative stress, and the improvement of specialized physiological functions, including reproductive health [8]. Probiotics provide benefits by essentially preventing the colonisation of pathogenic microorganisms in the gut, promoting the production of short-chain fatty acids, increasing the bioavailability of certain nutrients, supporting the immune system, and maintaining the integrity of the intestinal barrier [9]. These microorganisms can be naturally ingested through traditional fermented foods (such as yoghurt, kefir, and pickles); they can also be consumed through functional foods containing probiotics (such as probiotic yoghurts, drinks, and snacks) or commercial supplements in capsule, powder, and liquid forms [10]. Today, probiotic-containing foods and supplements have become a significant focus of interest, alongside increasing health awareness and changes in dietary behaviour. The global market value of probiotic-containing products is reported to have reached 87.7 billion US dollars in 2023 [11]. The frequency of consumption and reasons for choosing these foods and products are influenced by numerous factors, including sociodemographic characteristics, economic status, educational level, health perceptions, dietary habits, and knowledge of microbiota and probiotics [12, 13]. In particular, the concept of ‘microbiota awareness’ refers to the knowledge, attitudes, and behaviours related to the role of gut microbiota in human health, the importance of microbial balance, and the effects of probiotics and prebiotics. A limited number of studies conducted in recent years have shown that individuals with high levels of microbiota awareness exhibit healthier eating behaviours, consume foods containing probiotics and prebiotics more consciously, and generally adopt healthier lifestyle behaviours [14, 15]. However, studies examining the direct relationship between microbiota awareness levels and gastrointestinal health are quite limited in the literature. Similarly, the consumption of probiotic-containing foods has been reported to have potential beneficial effects in the management of gastrointestinal symptoms such as dyspepsia, constipation, diarrhoea, and abdominal pain. Nevertheless, the association between probiotic consumption frequency and gastrointestinal symptoms may be modulated by individual factors such as age, gender, dietary habits, and gut microbiota composition, and the literature shows heterogeneity in the results regarding this interaction [16, 17]. In this regard, utilizing validated tools such as the Gastrointestinal Symptom Rating Scale (GSRS) is important to correctly identify the severity of functional complaints in different groups [18, 19]. While the GSRS is widely used to monitor diagnosed patients in clinical practice, current research also highlights its value in evaluating the symptom burden in individuals who have functional discomfort but do not have a formal medical diagnosis [20, 21]. Determining the severity of symptoms in such groups is especially useful for identifying people who may benefit from early dietary awareness and probiotic use.

Although there are studies in the literature examining the relationship between the frequency of consumption of probiotic-containing foods and gastrointestinal symptoms, studies investigating the potential role of microbiota awareness in this relationship are limited. It is conceivable that individuals with a high level of awareness about the microbiota may consume probiotic-containing foods more regularly and may be more likely to adopt dietary and lifestyle behaviours that support gut health in general. Furthermore, identifying the reasons for consuming/not consuming probiotic-containing foods, information sources, and consumption habits within the community is important for developing health policies and educational strategies in this area. In this context, the main objective of the present study is to examine the relationship between the level of microbiota awareness and the frequency of probiotic-containing food consumption in adults and the severity of gastrointestinal symptoms. The study also aims to evaluate participants' probiotic consumption status, information sources on this topic, reasons for consuming or not consuming probiotic-containing foods, and the potential associations between sociodemographic, anthropometric characteristics, and gastrointestinal symptoms.

## Methods

### Participants and procedure

This cross-sectional study included adults aged 19–65 years. Sample size was determined based on a prior study [22]. Assuming an effect size of 0.552 with 95% confidence and 95% power, at least 176 participants were required. In total, 642 individuals (201 men, 441 women) completed an online survey. Participants were recruited using the snowball sampling technique via an online survey (Google Forms). The survey form was initially shared via social media platforms (Instagram, Twitter, Facebook) and the researchers' email lists, and participants were then asked to share the survey with suitable individuals in their circles. Eligible individuals aged 18–65 who provided informed consent and completed the survey in full were included. Participants approved the informed consent form at the beginning of the survey. Participants were excluded if they had a physician-diagnosed chronic gastrointestinal disease (irritable bowel syndrome, Crohn's disease, coeliac disease, ulcerative colitis, etc.), had used antibiotics or probiotic supplements in the last month, had a chronic disease requiring regular medication, or had a history of diagnosed eating disorder (anorexia nervosa, bulimia nervosa, binge eating disorder, etc.), a history of neurological disease that could affect gastrointestinal system functions (Parkinson's disease, multiple sclerosis, etc.), or a history of psychiatric disease requiring psychiatric medication were excluded from the study. Participants meeting any of the exclusion criteria were automatically filtered out via pre-questions at the start of the survey and were not included in the study.

Ethical approval was obtained from Ankara Medipol University Non-Interventional Ethics Committee on 27.05.2024 with decision number 67. Participation was voluntary, and informed consent was secured.

### Study design

The survey included demographic information, self-reported anthropometrics, probiotic food consumption habits, the Gastrointestinal Symptom Rating Scale (GSRS), and the Microbiota Awareness Scale (MAS).

### The Gastrointestinal Symptom Rating Scale (GSRS)

The Gastrointestinal Symptom Rating Scale (GSRS), developed by Revicki et al. [23] and validated in Turkish by Turan et al. [24], was used in the study to assess gastrointestinal symptoms. The scale consists of five subscales: abdominal pain, reflux, diarrhoea, indigestion, and constipation, and contains a total of 15 items. Each item is rated on a 7-point Likert scale, ranging from 1 ‘no discomfort’ to 7 ‘very severe discomfort’. The total scores range from 15 to 105, with higher scores indicating more severe gastrointestinal symptoms. The Cronbach's alpha reliability coefficient for the scale in the original study was reported as 0.89, while in the Turkish version it was found to be 0.92.

### Microbiota Awareness Scale (MAS)

The Microbiota Awareness Scale (MAS), developed by Külcü and Önal [25], was used to determine participants' levels of awareness regarding gut microbiota. The scale consists of a total of 20 items and is rated on a 5-point Likert scale (1 = Strongly disagree, 5 = Strongly agree). The total scores range from 20 to 100, with higher scores reflecting a higher level of awareness regarding the microbiota. The Cronbach's alpha reliability coefficient of the scale in the original study was reported as 0.87.

### Probiotic food consumption frequency

The consumption frequency of various foods containing probiotics (including yoghurt, kefir, and other fermented products) over the past month was assessed. For each food type, participants reported their consumption frequency on an 8-point ordinal scale (ranging from 0 = Never to 7 = More than once daily). A total frequency score was calculated by summing responses across all food types to reflect overall habitual intake.

### Body Mass Index (BMI)

Participants' body mass indices were calculated using their self-reported height and weight values. BMI was obtained by dividing body weight (kg) by height squared (m^2^) (kg/m^2^).

### Statistical analysis

Data were analyzed using SPSS 23.0 (IBM Corp., Armonk, NY). Descriptive statistics were expressed as frequency, percentage, mean, and SD. Pearson χ^2^ or continuity correction tested associations between categorical variables. Pearson correlation examined relationships among scales. Hierarchical multiple regression assessed the associations between microbiota awareness and probiotic food consumption frequency and gastrointestinal (GI) symptom severity through four models. The assumptions of the regression analysis (normality and homoscedasticity) and the presence of influential outliers were verified by inspecting the distribution of residuals. Multi-collinearity was monitored using Variance Inflation Factor (VIF), with all values remaining well below the threshold of 10. Since the rate of missing data was negligible, a complete-case analysis was used. In all hierarchical models, probiotic consumption frequency was treated as a continuous predictor to test for a linear trend. Model 1 was the crude model, Model 2 adjusted for age and BMI, Model 3 further adjusted for gender. Model 4 included educational status, employment status, income status, and the presence of digestive system problems as additional covariates. These variables were selected based on their availability within the study’s survey and as they represent key socioeconomic and clinical determinants that can independently influence both health awareness and gastrointestinal symptom reporting. Specifically, educational status, employment status, and income were included as proxies for socioeconomic position, which is known to influence both health behaviors and gastrointestinal symptom burden. The presence of digestive system problems was included as a clinically relevant confounder, given its direct relationship with GSRS scores. The inclusion of these specific variables aimed to provide a comprehensive adjustment for sociodemographic and baseline health factors available in our dataset. Other potentially relevant factors such as overall diet quality, psychological distress, physical activity, and medication use were not included in the regression models because these variables were not assessed within the survey design. Therefore, adjustment was restricted to the predefined variables collected in the dataset.

## Results

Table 1 presents participants’ general characteristics and scale scores by probiotic consumption status. Women reported more frequent probiotic food consumption than men (*p* < 0.001). Individuals with higher income also consumed probiotics more frequently (*p* < 0.001).

**Table 1:** General characteristics and scale scores of regular probiotic-containing foods consumers and non-regular consumers

Variables	Regular consumers (*n* = 503)	Non-regular consumers (*n* = 139)	Total (*n* = 642)	p*
Age (X̄ ± SD)	34.4 ± 11.41	30.7 ± 10.97	33.5 ± 11.40	< 0.001
Gender, n (%)				
Male	138(27.4)	63(45.3)	201(31.3)	<0.001
Female	365(72.3)	76(54.7)	441(68.7)	
Educational status, n (%)				
Primary school	3(0.6)	1(0.7)	4(0.6)	0.414
High school	45(8.9)	17()12.2	62(9.7)	
Bachelor's degree and above	455(90.5)	121(87.1)	576(89.7)	
Employment status, n (%)				
Employed	341(67.8)	86(61.9)	427(66.5)	0.227
Not employed	162(32.2)	53(38.1)	215(33.5)	
Income status, n (%)				
Less than expenses	104(20.7)	45(32.4)	149(23.2)	0.011
Equal than expenses	236(46.9)	60(43.2)	296(46.1)	
More than expenses	163(32.4)	34(24.5)	197(30.7)	
Digestive system problem, n (%)				
None	323(64.2)	102(73.4)	425(66.2)	0.101
Stomach disease	88(17.5)	24(18.0)	113(17.6)	
Intestinal disease	85(16.9)	11(7.9)	96(15.0)	
Bloating-gas problem	7(1.4)	1(0.7)	8(1.2)	
Body weight (X̄ ± SD)	70.6 ± 14.31	71.2 ± 16.09	70.7 ± 14.70	0.689
BMI (X̄ ± SD)	24.5 ± 4.45	25.1 ± 4.28	24.9 ± 4.32	0.207
MAS (X̄ ± SD)	67.5 ± 14.53	61.4 ± 12.25	66.2 ± 14.28	**< **0.001
GSRS total score (X̄ ± SD)	31.3 ± 14.93	31.3 ± 15.23	31.3 ± 15.76	0.929

Data were presented as mean ± standard deviation for continuous variables and n (%) for categorical variables. *p-values were obtained Independent-Samples t-test, Pearson χ^2^ test and Continuity Corretion, as appropriate

*BMI* Body mass index, *MAS* Microbiota Awareness Score *GSRS* Gastrointestinal Symptom Rating Scale, *SD* Standard deviation

**Fig. 1:**
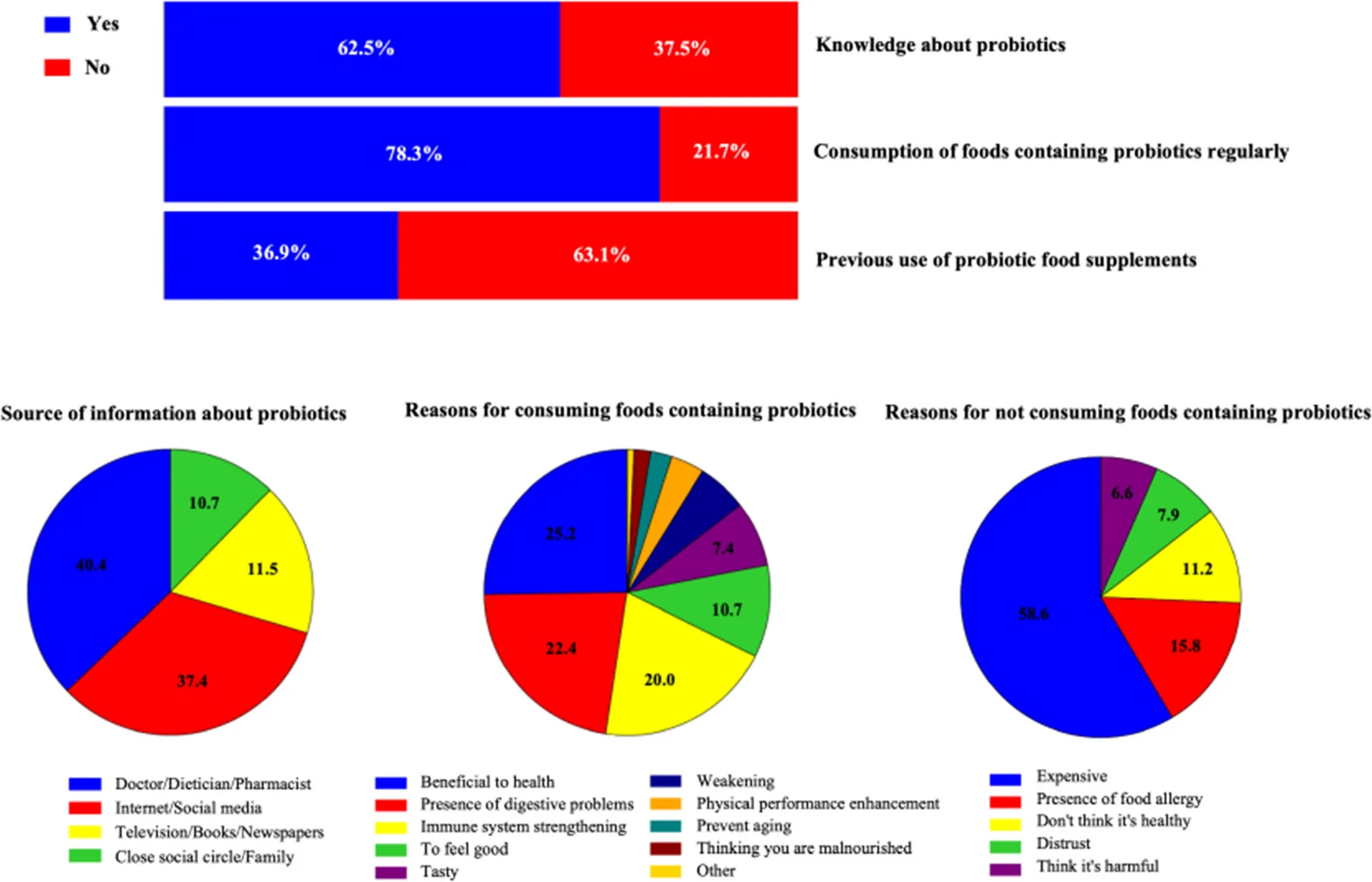
Probiotic Consumption Status, Information Sources and Reasons for Use/Non-use

Figure 1 illustrates that 62.5% of participants reported being knowledgeable about probiotics, and 78.3% regularly consumed probiotic foods. The most common information sources were health professionals (40.4%) and the internet/social media (37.4%).

Table 2 shows correlations between probiotic consumption frequency and scale scores. MAS scores were negatively correlated with GSRS scores (r = −0.268, *p* < 0.001), indicating that higher microbiota awareness was associated with lower GI symptom severity. GSRS and probiotic consumption frequency correlated negatively but not significantly (r = −0.040, *p* > 0.05). MAS and probiotic consumption frequency correlated positively and significantly (r = 0.085, *p* < 0.05).

**Table 2:** Relationships between GSRS, microbiota awareness level scale and probiotic-containing food consumption frequency (r)

		**GSRS Subscales**							
		**1**	**2**	**3**	**4**	**5**	**6**	**7**	**8**
GSRS Subscales	**1. Abdominal Pain**	-							
	**2. Reflux Syndrome**	0.692^**^	-						
	**3. Diarrhea Syndrome**	0.564^**^	0.508^**^	-					
	**4. Indigestion Syndrome**	0.702^**^	0.537^**^	0.572^**^	-				
	**5. Constipation Syndrome**	0.553^**^	0.431^**^	0.557^**^	0.648^**^	-			
	**6. GSRS Total Score**	0.849^**^	0.727^**^	0.773^**^	0.889^**^	0.808^**^	-		
	**7. Microbiota awareness**	−0.269^**^	−0.199^**^	−0.186^**^	−0.241^**^	−0.192^**^	−0.268^**^	-	
	**8. Probiotic-containing food consumption frequency**	−0.043	−0.024	0.044	−0.076	−0.036	−0.040	0.085^*^	-

Pearson correlation. r:correlation coefficient

^*^p < 0.05, **p < 0,001

*GSRS* Gastrointestinal Symptom Rating Scale

Hierarchical regression results are summarized in Table 3. In Model 1, microbiota awareness showed a significant association with GI symptom severity (β = −0.266, *p* < 0.001), explaining 7.2% of the total variance. In Model 2, age and BMI were added but did not significantly improve the model fit (ΔR^2^ = 0.004, *p* = 0.235). In Model 3, gender was included and significantly increased the explained variance to 11.4% (ΔR^2^ = 0.038, *p* < 0.001); awareness remained significant (β = −0.276, *p* < 0.001), BMI showed a positive association (β = 0.085, *p* = 0.035), and gender was a significant correlate (B = 6.802, *p* < 0.001), while age did not reach statistical significance (β = −0.065, *p* = 0.101). Finally, in Model 4, educational status, employment, income, and digestive system problems were added, resulting in a substantial increase in the explanatory power of the model (R^2^ = 0.254, Adj. R^2^ = 0.243). This final model explained 25.4% of the total variance in GI symptom severity, representing a significant improvement over Model 3 (ΔR^2^ = 0.139, F = 29.514, *p* < 0.001). In this fully adjusted model, microbiota awareness remained a strong independent correlate of lower GI symptom severity (β = −0.288, *p* < 0.001), while older age (β = −0.137, *p* = 0.001), educational status (B = −3.894, *p* = 0.035) and having a digestive system problem (B = 12.313, *p* < 0.001) were also identified as significant associated factors.

**Table 3:** Associations of microbiota awareness, probiotic-containing food consumption frequency, and selected variables with GSRS scores

Model	Variables	B	β	SE	t	p	95% CI		VIF
1	(Constant)	51.199		2.932	17.462	< 0.001	45.441	56.956	
	Microbiota awareness	−0.294	−0.266	0.042	−6.959	< 0.001	−0.377	−0.211	1.007
	Probiotic-containing food consumption frequency	−0.020	−0.018	0.044	−0.464	0.643	−0.106	0.065	1.007
2	(Constant)	49.822		4.555	10.939	< 0.001	40.878	58.766	
	Microbiota awareness	−0.290	−0.263	0.042	−6.863	< 0.001	−0.373	−0.207	1.010
	Probiotic-containing food consumption frequency	−0.015	−0.013	0.044	−0.342	0.732	−0.102	0.071	1.033
	Age	−0.089	−0.064	0.056	−1.585	0.113	−0.199	0.021	1.133
	BMI	0.160	0.044	0.147	1.089	0.276	−0.129	0.449	1.120
3	(Constant)	42.783		4.662	9.177	< 0.001	33.628	51.937	
	Microbiota awareness	−0.305	−0.276	0.042	−7.345	< 0.001	−0.386	−0.223	1.015
	Probiotic-containing food consumption frequency	−0.032	−0.028	0.043	−0.737	0.461	−0.117	0.053	1.039
	Age	−0.090	−0.065	0.055	−1.643	0.101	−0.198	0.018	1.133
	BMI	0.311	0.085	0.147	2.116	0.035	0.022	0.599	1.165
	Gender	6.802		1.301	5.229	< 0.001	4.247	9.356	1.053
4	(Constant)	43.647		4.402	9.914	< 0.001	35.002	52.293	
	Microbiota awareness	−0.318	−0.288	0.038	−8.280	< 0.001	−0.394	−0.243	1.028
	Probiotic-containing food consumption frequency	0.006	0.006	0.040	0.159	0.874	−0.073	0.085	1.057
	Age	−0.190	−0.137	0.055	−3.476	0.001	−0.297	−0.083	1.322
	BMI	0.274	0.075	0.136	2.019	0.044	0.007	0.540	1.169
	Gender	5.687		1.234	4.607	< 0.001	3.263	8.111	1.118
	Educational status	−3.894		1.847	−2.109	0.035	−7.521	−0.268	1.073
	Employment status	0.505		1.287	0.392	0.695	−2.023	3.032	1.259
	Income status	1.383		1.310	1.056	0.292	−1.190	3.956	1.044
	Digestive system problem	12.313		1.187	10.375	< 0.001	9.983	14.644	1.075

Model summary: Model 1 (R^2^ =.072, F_change_ = 24.770, *p* <.001); Model 2 (R^2^ =.076, ΔR^2^ =.004, F_change_ = 1.454, *p* =.235); Model 3 (R^2^ =.114, ΔR^2^ =.038, F_change_ = 27.340, *p* <.001); Model 4 (R^2^ =.254, ΔR^2^ =.139, F_change_ = 29.514, *p* <.001; Adjusted R^2^ =.243). Hierarchical multiple regression analysis. Model 1: Crude, Model 2:Age and BMI, Model 3:Age, BMI and gender, Model 4: Educational status, Employment status, Income status and Digestive system problem. Binary/Dummy variables were coded as follows: Gender (0 = Male, 1 = Female), Educational status (0 = Bachelor's degree and above, 1 = Below Bachelor's degree), Employment status (0 = Employed, 1 = Not employed), Income status (0 = Equal/More than expenses, 1 = Less than expenses), Digestive system problem (0 = None, 1 = At least one diagnosed problem). GSRS: Gastrointestinal Symptom Rating Scale, B: Unstandardized coefficient, β: Standardized coefficient, SE: Standard error, CI: Confidence interval, VIF: Variance inflation factor; BMI: Body mass index

## Discussion

This study examined probiotic food consumption patterns and their associated factors, as well as the relationships between microbiota awareness, probiotic food consumption frequency, and gastrointestinal symptom severity, including the potential contributions of sociodemographic and clinical parameters. The findings revealed substantial variation in probiotic food consumption and the motivations underlying its use. In addition, it demonstrated significant relationships between microbiota awareness and the frequency of probiotic food consumption. The level of microbiota awareness, in particular, was found to be significantly associated with gastrointestinal symptom severity. To our knowledge, this study is one of the limited studies that comprehensively evaluates probiotic-containing foods, consumption frequencies, microbiome awareness levels, and GI symptom severity.

Today, the interest in functional food consumption has risen alongside the increasing desire for a healthy lifestyle [26]. Since probiotic foods constitute a significant part of the functional food market, they are among the most preferred functional foods by consumers both in the world and in our country [26, 27]. In our study, it was determined that 62.5% of participants were knowledgeable about probiotics, and 78.3% regularly consumed probiotic foods. A similar study conducted in our country also revealed that the participants were highly aware of the probiotic food consumption benefits, and a significant majority (66.8%) were willing to purchase probiotic products on the market [26]. In contrast, Aslan et al. [28] found that 26% of the participants preferred to consume probiotic products. These different results may be linked to factors such as education levels, economic income, and sources of information. Aslan et al. [28] also reported that probiotic food consumption preferences correlated positively with individuals' education and income levels. In our study, the most common reason stated by individuals who do not consume probiotic foods is the concern about high prices (58.6%). This result also reveals that economic income is associated with the consumption of probiotic foods. In addition, the study found that probiotic foods are preferred mainly because they are beneficial to health, eliminate digestive system problems, and strengthen the immune system. These results are also similar to those in the literature. Many studies have stated that probiotic foods are consumed due to their general health benefits, especially for the digestive system, and the desire to maintain a healthy lifestyle [26–29].

It is important to note that our participants predominantly represent highly educated and digitally connected demographic subgroup, with approximately 90% holding a university degree. While this is significantly higher than the 44.9% tertiary education rate reported by Turkish Statistical Institute (TurkStat) for the 25–34 age group [30], it allows for a focused look at a subpopulation with high health literacy. This educational tilt likely introduces a positive bias, meaning the awareness and consumption levels found in this study may be higher than those of the general public. Thus, our findings should be viewed as reflective of this specific educated urban group rather than a direct representation of the entire Turkish population. Furthermore, participants were recruited online through social media and researcher networks, which likely favored individuals with greater digital access, higher health awareness, and stronger engagement with health-related content. Consistent with this profile, our study demonstrated that the most common information sources about probiotic foods were health professionals (40.4%), followed by digital sources (37.4%). This finding indicates that highly educated individuals regard health professionals as reliable sources of information on nutrition and gut health. The literature also supports the tendency of highly educated individuals to turn to professional sources on health-related issues. In a study conducted with approximately 700 highly educated participants, it was reported that the most common source of information about probiotics was health professionals, and 71.5% of the participants were willing to use probiotics if recommended by a health professional [31]. Social media and the internet are among the most common information sources about probiotic foods, supporting the fact that digital sources are increasingly used to access nutritional information. However, there are concerns about the reliability and accuracy of these sources [32]. Previous studies have emphasized that digital sources can effectively reach a broad audience with nutritional information but that the scientific accuracy of the content should be ensured [33, 34]. In this study, it can be thought that individuals with a higher level of education may be more sensitive in referencing scientific data by using digital sources with a critical perspective. However, it is important for public health that health professionals take a more active role in digital platforms and disseminate scientifically based information on these channels.

Microbiota awareness refers to the comprehension and knowledge that individuals have about the effects of microbiota on health [35]. Understanding the interactions between intestinal microbiota and health can enable individuals to make conscious and healthier lifestyle choices that positively affect their intestinal bacterial composition [14, 36]. Previous research consistently demonstrates that higher levels of microbiota and health awareness are associated with increased consumption of probiotics, prebiotics, and fermented foods [22, 29, 31, 37, 38]. Consistent with these findings, our study found a significant positive relationship between microbiota awareness and probiotic food consumption frequency (Table 2). This finding suggests that a higher level of microbiota awareness is linked to a higher frequency of consuming probiotic-containing foods. In contrast, Alqurashi et al. [39] found that individuals who were unfamiliar with the terms “prebiotic” and “probiotic” had relatively high consumption of pre/probiotic products. This situation may be related to the culinary culture of individuals. The prevalent intake of health-promoting products in that culture, particularly fermented foods, may be associated not only with the degree of knowledge and awareness but also with established habits [38]. Consequently, the link between microbiota awareness and the consumption of microbiome-friendly foods is complex and influenced by various factors, including demographics.

One of the important results obtained in the study is the association between individuals' microbiota awareness levels and GI symptom severity. A negative correlation was found between the microbiota awareness levels of individuals and the GSRS scores, indicating GI symptom severity (*p* < 0.001) (Table 2). In addition, the association between microbiota awareness and GI symptom severity were found to be significant in all models in further analyses (*p* < 0.001) (Table 3). The results indicated that higher microbiota awareness is associated with lower GI symptom severity, a finding that remained significant even when variables such as age, BMI, and gender were controlled. In the final model, educational status, employment status, and income were additionally included as covariates given that lower socioeconomic position has been shown to independently associate with higher gastrointestinal symptom burden in large population-based studies [40]. The presence of digestive system problems was also included as a covariate, as individuals with pre-existing gastrointestinal conditions are known to report significantly higher GSRS scores compared to those without such conditions [23]. Despite this comprehensive adjustment, microbiota awareness remained a significant independent correlate of GI symptom severity, strengthening the robustness of the findings. Clinically, the final model indicates that every 10-point increase in the MAS was linked to an approximately 2.9 unit decrease in the GSRS total score. This suggests that even incrementally higher levels of gut health awareness are associated with a lower subjective burden of functional gastrointestinal symptoms. This relationship may be explained by 'preventive health behaviors,' as individuals with higher awareness are more likely to prioritize dietary fiber diversity and avoid pro-inflammatory patterns. Such conscious nutritional choices are known to stabilize the intestinal environment and modulate the gut-brain axis, which is linked to lower both the physiological and perceived severity of gastrointestinal discomfort [14].

The Gastrointestinal Symptom Rating Scale (GSRS) is a widely used tool for assessing the severity of gastrointestinal symptoms. In our study, participants were individuals with functional symptoms but no formal medical diagnosis. The mean GSRS score of 31 in this group reflects a symptomatic burden that is higher than scores found in healthy athletes [21] but remains below the levels seen in medication-induced intolerance [19]. It has been used in several studies to evaluate the potential relationship between fermented foods containing probiotics and GI health. Urita et al. [41] discovered that drinking fermented milk with Bifidobacterium bifidum lowered GSRS scores in patients with functional GI disorders. Another study reported that after consuming a fermented product containing probiotics for 3 weeks, people experienced decreased GI complaints, GSRS scores, and significant increases in bifidobacterium levels in their fecal microbiota [42]. In addition, there are studies evaluating the potential relationship between probiotic supplement use and GSRS [17, 43]. However, these studies did not evaluate food consumption frequencies. In our study, a negative relationship was found between the frequency of probiotic food consumption and GSRS scores (Table 2). This suggests a potential inverse relationship, where a higher frequency of probiotic food consumption is associated with lower GIS severity. However, this relationship was not found to be statistically significant. In addition, no significant differences were found in GSRS scores between individuals who regularly consumed probiotic foods and those who did not (Table 1). In addition, the associations between the frequency of probiotic food consumption and GSRS were not significant in the regression model (Table 3). These results may be due to the fact that only probiotic food consumption was evaluated in the study, and prebiotic foods or general dietary patterns were not evaluated. Furthermore, our assessment of probiotic intake was limited to consumption frequency. The absence of a significant association between probiotic food consumption and GI symptom severity in the regression model may also be attributed to unmeasured variables such as the specific probiotic strains, the exact quantity (dosage), the type of product, and the total duration of intake. In the literature, the therapeutic effects of probiotics on GI health are often dose- and strain-specific [44, 45]. Therefore, without accounting for these qualitative and quantitative factors, the independent association of probiotic food consumption with GSRS scores may not have been fully captured in our study. Considering the role of probiotic foods in intestinal microbiota, the association of these foods with GI symptom severity may become more apparent together with prebiotics or other nutritional factors [46, 47]. Future studies should evaluate probiotic foods, prebiotic intake, and overall dietary composition together. Furthermore, it is important to note that this study did not assess general dietary patterns, prebiotic intake, psychological status, or physical activity levels. These factors are well-documented to influence gastrointestinal symptom severity and microbiota composition [46–52]. As these variables were not included in our analysis, they may serve as potential confounders, and their absence should be considered when interpreting the relationships between microbiota awareness, probiotic consumption, and GI health. In particular, diet quality represents a critical unmeasured confounder since individuals with higher microbiota awareness may adhere to more diverse and fiber rich dietary patterns. Such habits are independently associated with lower gastrointestinal symptom severity and a more favorable gut microbiota composition [50]. If this is the case, the observed MAS-GSRS association may partly reflect the influence of underlying diet quality rather than awareness itself. Similarly, psychological distress including anxiety and depression is well documented to exacerbate functional gastrointestinal symptoms through gut brain axis dysregulation [49, 51]. Since psychological status was not assessed in this study, its potential confounding role cannot be excluded. Physical activity is another relevant factor because regular exercise is associated with both improved gastrointestinal motility and higher gut microbial diversity, and individuals with greater health awareness may also be more physically active [52]. Finally, health literacy is a broader construct that likely encompasses microbiota awareness. Beyond specific knowledge of probiotics, it extends to general self-care behaviors, medication adherence, and preventive health engagement, all of which may independently influence symptom reporting patterns. Because the MAS captures awareness specifically related to the microbiota, it may partially serve as a proxy for this wider health literacy, making these overlapping constructs difficult to distinguish in a cross-sectional self-report design. Consequently, future research should incorporate validated measures of diet quality, psychological status, and general health literacy to more precisely isolate the independent contribution of microbiota awareness to gastrointestinal health.

When other variables that may be associated with GI symptom severity were examined, it was determined that gender and BMI also showed significant associations with GI symptom severity (Table 3). Women may be more affected due to psychological factors and hormonal status, both of which exacerbate functional GI disorder symptoms [51, 53].

Furthermore, when the relationships between BMI and GI symptom severity were examined, previous studies demonstrated a correlation between high BMI values and increased GI symptom severity [54–56]. Huseini et al. [54] showed that the frequency of GI symptoms (dysphagia, gastroesophageal reflux, abdominal pain) is higher in individuals with class III obesity compared to those without obesity and that high BMI levels are associated with increased GI symptom severity and frequency. Another study reported that individuals with obesity have higher constipation (87.9%) and abdominal distension (83.3%) compared to normal weight individuals [55]. In conclusion, our findings align with the literature, showing that women and individuals with higher BMI levels experience greater GI symptom severity. In terms of clinical practice, these findings emphasize that enhancing 'gut health literacy' may be considered as an integral part of patient education in gastrointestinal management. Rather than solely focusing on specific probiotic prescriptions, healthcare professionals particularly dietitians can leverage microbiota awareness as a strategic, low-cost, non-pharmacological tool. Empowering patients to understand the symbiotic relationship between diet and microbiota is associated with more sustainable, health-conscious choices, which may be linked to a lower subjective burden of functional GI symptoms.

This study had some limitations. First of all, since the study had a cross-sectional design, it could not reveal a cause-and-effect relationship. This design also introduces the possibility of reverse causality, where individuals with fewer gastrointestinal symptoms might be more inclined to develop higher microbiota awareness. Furthermore, unmeasured factors such as general health literacy or health-seeking behaviors may independently influence both awareness levels and symptom reporting. Although the results were sufficient to explain the relationship between the variables, the potential for longitudinal or temporal relationships could not be evaluated. Future prospective studies can provide a better understanding of the relationships between the variables. Another limitation is that individuals' probiotic food consumption was evaluated only on a frequency basis; portion sizes or quantitative intake data were not collected. Critically, no detailed information was gathered regarding the specific probiotic strains, the type of products consumed, or the long-term duration of intake. Given that the health benefits of probiotics are highly dependent on the specific strain and an adequate 'dose' over time, the lack of these parameters limits the clinical interpretation of our findings regarding probiotic efficacy. Future prospective studies should utilize comprehensive dietary assessment tools to capture these quantitative and qualitative variables in more detail. Furthermore, the gender distribution of our study population was highly imbalanced, with women representing 68.7% of the participants. This gender disproportion limits the generalizability of the findings to the broader population, particularly to men, and should be considered when interpreting the results.

It is important to emphasize that this study best reflects a health-literate, internet-recruited adult subgroup rather than the general Turkish population. The online snowball recruitment strategy preferentially attracted younger, urban, digitally literate, and health-conscious individuals with higher levels of education. Consequently, these findings may not be fully representative of the broader public, particularly those with limited educational access or digital engagement.

In addition, general dietary patterns, prebiotic intake, psychological status, physical activity, and medication use were not assessed. Specifically, the lack of a comprehensive dietary assessment (e.g. Food Frequency Questionnaire for fiber or FODMAP load) is a significant limitation, as these factors may confound or mediate the observed MAS–GSRS association. Furthermore, the MAS should be considered a proxy marker of gut health literacy rather than a direct measure of microbial health. These unmeasured confounders should be considered when interpreting the results. Furthermore, the study relied entirely on self-reported data (BMI, GI symptoms, and consumption habits), which may introduce reporting bias and should be considered when interpreting the objectivity of the results. Despite these limitations, the study also has some strengths. One of its most important strengths is that it has an approach that can contribute to previous studies by evaluating associations between microbiota knowledge and awareness with GI symptom severity. To the best of our knowledge, the associations between microbiota awareness, probiotic food consumption, and GI symptom severity have not been examined in any previous study. In addition, the regression models constructed in the study helped determine the independent associations of microbiota awareness level and probiotic food consumption with GI symptom severity, and the role of potential confounding factors such as gender, age, and BMI were also analyzed.

## Conclusion

The findings from this study indicate that the level of microbiome awareness is a significant correlate of gastrointestinal symptom severity. The findings support the hypothesis that higher microbiota awareness may reflect healthier symptom-related behaviors and greater gut health literacy. However, they should not be interpreted as evidence of a therapeutic effect or causal benefit of microbiota awareness on gastrointestinal symptoms. In contrast to awareness levels, no significant independent association was observed between probiotic-containing food consumption frequency and gastrointestinal symptom severity in the adjusted models. These findings should primarily be interpreted within the context of a digitally recruited, predominantly female, urban, and highly educated subgroup of Turkish adults. Furthermore, to broaden these insights, a more detailed examination of factors such as the strain type, dosage, consumption duration, and individual differences of probiotic-containing foods is warranted. In addition to probiotic-containing foods, it will also be important to evaluate general dietary habits, prebiotic intake, psychological status, and physical activity levels, all of which are associated with the microbiota and gastrointestinal health. Furthermore, variables such as BMI and gender have also been identified as factors associated with the severity of gastrointestinal symptoms. The associations between these factors and gastrointestinal health should also be considered. Longitudinal and intervention-designed studies are needed to evaluate the relationship between probiotic-containing foods and gastrointestinal health in greater detail.

## Data Availability

The datasets are available from the corresponding author on reasonable request.

## Abbreviations

GSRS: Gastrointestinal Symptom Rating Scale
MAS: Microbiota Awareness Scale
GI: Gastrointestinal
BMI: Body Mass Index
